# Evaluation of patients with angioedema in accompaniment in the allergy and immunology HSPE / SP

**DOI:** 10.1186/1939-4551-8-S1-A233

**Published:** 2015-04-08

**Authors:** Marilise Marculino, Chayanne Andrade Araújo, Dayane Brandini, Wilson Tartuce Aun, João Ferreira Mello, Fatima Rodrigues Fernandes, Maria Elisa Andrade, Paula Sá Barreto, Camila Aparecida Campos Teixeira

**Affiliations:** 1 Hospital Do Servidor Publico Estadual De São Paulo, Brazil

## Background

Angioedema is defined as delimited edema that compromises dermis and subcutaneous. May have different causes hypersensitivity, autoimmune, idiopathic, physical factors, angiotensin-converting enzyme inhibitors and C1 esterase inhibitor consumption or disability. The physiopathologic mechanisms depend on the cause. In addition to detailed history, additional tests are needed for targeting of etiology. Our Objective was evaluate the profile of patients treated in outpatient Angioedema Allergy and Immunology HSPE.

## Methods

We included patients from the Allergy and Immunology Service, attended between January 2013 and June 2014, with angioedema as the main symptom. The following tests were ordered: Blood count, IgE, TSH, FT4, anti-TPO and anti-TG, ANA, RF, complement (C3, C4, C2, CH50, C1q, C1 esterase inhibitor), CEA, CA 125, CA 19.9 and PSA. In addition, research allergic (immediate skin test, patch test, specific IgE test for physical urticaria, drug provocation when necessary) was also performed.

## Results

Of the 110 patients with a mean age of 54,4 years, 80% was female. Only 20% have a family history of angioedema and 42,7% have a personal history of atopy. Among the possible triggering, ACE inhibitors and NSAIDs and were the most prevalent, accounting for 20,9% and 29,1% respectively, followed by ARB (8,2%), food (8,2%), contactants (7,3%) and others (2,7%). Patients under investigation and no defined causes account for 22,7%.

## Conclusions

We observed in this sample that the drugs were the most common triggers of angioedema, with greater relevance to the association with NSAIDs and ACE inhibitors. Patients with urticaria were mainly associated triggering the use of NSAIDs, while in cases associated with ACE inhibitors did not observe reports of urticaria.

